# Presenteeism Among Health Care Personnel With COVID-19

**DOI:** 10.1001/jamanetworkopen.2025.46405

**Published:** 2025-12-03

**Authors:** James C. Crosby, Eliezer Santos Leon, Brian Chinnock, Karisa K. Harland, Anusha Krishnadasan, Nicholas M. Mohr, Ian D. Plumb, Melissa Briggs Hagen, Kelli Wallace, David A. Talan, and the Project PREVENT Network, Anne Zepeski, Tracy Young, Howard A. Smithline, Lilly C. Lee, Stephen C. Lim, Gregory J. Moran, Mark T. Steele, David G. Beiser, John P. Haran, Peter C. Hou, Brett Faine, Utsav Nandi, Walter A. Schrading, Anne Chipman, Frank LoVecchio, Alysia Powers, Lisandra Uribe, Kavitha Pathmarajah, Dean M. Hashimoto, Chloe Namias, Efrat Kean, Elizabeth Krebs, Amy Stubbs, Sara Roy, Lucia Solis, Mary Mulrow, Nathan Graff, Jillian M. Tozloski, William Mower, Jacqueline Caldera, Michelle Huber, Jacob Hampton, Abigail Lopes, Katherine Elkort, Stephanie A. Eucker, Carla Kingsbury, Jonathan Femling, Silas Bussmann, Jane Yee, Joseph Stuppy, Richard E. Rothman, Gaby Dashler, Marcel E. Curlin, Mastura Wahedi, Laurie Kemble

**Affiliations:** 1 Department of Emergency Medicine, University of Alabama at Birmingham; 2 Department of Emergency Medicine, Carver College of Medicine, University of Iowa, Iowa City; 3 University of California-San Francisco Fresno School of Medicine, San Francisco; 4 Department of Epidemiology, College of Public Health, University of Iowa, Iowa City; 5 Olive View–University of California Los Angeles Education and Research Institute, Los Angeles; 6 Department of Anesthesia Critical Care, Carver College of Medicine, University of Iowa, Iowa City; 7 National Center for Immunizations and Respiratory Diseases, Centers for Disease Control and Prevention, Atlanta, Georgia; 8 University of Iowa Holden Comprehensive Cancer Center, University of Iowa, Iowa City; 9 Department of Emergency Medicine, David Geffen School of Medicine, University of California, Los Angeles; 10 Baystate Medical Center, Springfield, Massachusetts; 11 Brigham and Women’s Hospital, Boston, Massachusetts; 12 Centers for Disease Control and Prevention, Atlanta, Georgia; 13 Duke University School of Medicine, Durham, North Carolina; 14 Durham VA Health Care System, Durham, North Carolina; 15 Jackson Health System, Miami, Florida; 16 Jackson Memorial Hospital, Miami, Florida; 17 Johns Hopkins University, Baltimore, Maryland; 18 LSU Health New Orleans, New Orleans, Louisiana; 19 Olive View UCLA Medical Center, Los Angeles, California; 20 Oregon Health & Science University, Portland; 21 Johns Hopkins University School of Medicine, Baltimore, Maryland; 22 The University of Chicago, Chicago, Illinois; 23 University of Iowa, Iowa City; 24 University of Mississippi Medical Center, Jackson; 25 University of New Mexico Health Sciences Center, Albuquerque; 26 University of Utah School of Medicine, Salt Lake City; 27 Thomas Jefferson University, Philadelphia, Pennsylvania; 28 UCLA Emergency Medicine Center, Los Angeles, California; 29 University Health, Kansas City, Missouri; 30 University Medical Center New Orleans, New Orleans, Louisiana; 31 University of Alabama at Birmingham, Birmingham; 32 University of California Los Angeles David Geffen School of Medicine, Los Angeles; 33 Department of Medicine, University of Chicago, Chicago, Illinois; 34 University of Iowa Health Care, Iowa City; 35 University of Iowa Hospitals and Clinics, Iowa City; 36 University of Massachusetts Chan Medical School, Boston; 37 Department of Emergency Medicine, University of Massachusetts Chan Medical School, Boston; 38 University of Mississippi, Oxford; 39 School of Medicine, University of Missouri, Kansas City; 40 University of Washington School of Medicine, Seattle; 41 Valleywise Health Medical Center, Phoenix, Arizona

## Abstract

**Importance:**

Presenteeism—defined as continuing to work during an illness—poses a public health risk in the workplace and is especially hazardous within health care institutions where vulnerable patients may be exposed to nosocomial infections. Understanding the frequency and characteristics of health care personnel (HCP) who report presenteeism while ill with COVID-19 may help mitigate SARS-CoV-2 spread in hospitals and other health care institutions.

**Objectives:**

To determine the frequency of presenteeism among HCP with symptomatic COVID-19, and to evaluate the demographic, occupational, and clinical factors associated with it.

**Design, Setting, and Participants:**

This is an observational cohort study that uses data from the Preventing Emerging Infections Through Vaccine Effectiveness Testing (PREVENT) project: a test-negative, case-control vaccine effectiveness study that enrolled HCP who had COVID-19 symptoms at 24 academic medical centers from December 2020 through April 2024.

**Exposure:**

Exposures include demographic, occupational, and clinical characteristics of participants.

**Main Outcomes and Measures:**

Having confirmed symptomatic COVID-19 infection and reporting presenteeism; overall frequency of presenteeism through the study period and the association of the exposure characteristics with presenteeism, adjusting for confounders using 3 multivariable models. Presenteeism was defined as HCP who did not stop working during their illness, but the study did not differentiate whether they continued working remotely.

**Results:**

A total of 3721 HCP were included in the analysis (2842 [76.4%] aged 18-49 years; 2993 [80.4%] female; 278 [7.5%] Asian, 406 [10.9%] Black, and 2912 [78.3%] White). Overall, 293 (7.9%) reported presenteeism during the study period, and the frequency of presenteeism increased each year of the study period (from 1 of 73 [1.4%] in 2020 to 16 of 105 [15.2%] in 2024). Presenteeism was associated with HCP who have minimal patient contact (adjusted odds ratio [aOR], 3.73; 95% CI, 2.39-4.37), a graduate or professional degree (aOR, 1.90; 95% CI, 1.45-2.50), and income over $100 000 (aOR, 1.74; 95% CI, 1.12-2.69).

**Conclusion and Relevance:**

In this observational cohort study of 3721 HCP, there was an increasing frequency of presenteeism from 2020 through 2024, and job role and socioeconomic factors were associated. More studies are needed to understand the rationale behind the decision to continue working and the exact causes of presenteeism’s rising incidence among HCP with COVID-19.

## Key Points

**Question:**

What is the frequency of presenteeism among health care personnel (HCP) with symptomatic COVID-19, and which characteristics are associated with this behavior?

**Findings:**

In this cohort study of 3721 full-time HCP with symptomatic COVID-19 from 24 US academic medical centers, 7.9% reported presenteeism, with frequency increasing each year from 1.4% in 2020 to 15.2% in 2024. Presenteeism was significantly associated with minimal patient contact, graduate or professional degrees, and annual income over $100 000.

**Meaning:**

These findings suggest that presenteeism among HCP with COVID-19 increased over time and was associated with job roles and socioeconomic factors.

## Teaser

This cohort study evaluates the frequency of presenteeism among health care personnel with symptomatic COVID-19.

## Introduction

Presenteeism is defined as continuing to attend work during an illness.^1^ This is in contrast with absenteeism, when a person misses work due to an illness. Before the early 2000s, presenteeism was used primarily in business and social science literature to describe how individuals, when ill, lose the ability to function well at work and lose workplace productivity.^2^ In the last 2 decades, however, the term has evolved alongside recognition that presenteeism also poses a public health threat from the potential spread of pathogens in the workplace.^2^ This threat is a particular concern in health care institutions, where vulnerable patients may be exposed to ill and contagious health care personnel (HCP). Presenteeism among HCP has contributed to outbreaks of iatrogenic viral gastroenteritis and influenza in hospitals and long-term care facilities.^3,4^

The public health implications of presenteeism have never been more realized than during the COVID-19 pandemic. There are many examples of hospital and long-term care facility outbreaks that were traced back to HCP working with symptomatic COVID-19.^5,6,7^ Even in more recent years, when the severity of COVID-19 disease has decreased because of widespread immunity and weaker variants, the virus poses significant risk for immunocompromised and other high-risk patients in the hospital setting.^8^

We sought to determine the frequency and characteristics of presenteeism among HCP with symptomatic COVID-19 from December 2020 through April 2024, in particular those who continued working through the entirety of their known COVID-19 illness. The primary objectives of this study were to (1) determine the frequency of presenteeism among HCP through the study period and (2) identify characteristics associated with presenteeism compared with those who stopped working while ill.

## Methods

### Population

We performed an analysis of US HCP who were enrolled as part of a larger case-control vaccine effectiveness project, the Preventing Emerging Infections through Vaccine Effectiveness Testing (PREVENT) project, a test negative case-control study. In the PREVENT project, HCP from 24 participating health care systems were recruited if they tested for COVID-19 from December 2020 through April 2024. Participants completed a standardized enrollment survey 14 to 60 days after their test date that assessed information on demographic, occupational, and clinical characteristics, as well as whether they stopped working due to their illness. Specific details of the PREVENT study protocol are available online.^9^

In brief, all HCP included in PREVENT had SARS-CoV-2 diagnostic testing performed before enrollment into the project. All participants were either previously aware of or informed of the results of their COVID-19 test before participating in the study. Diagnostic tests included real-time polymerase chain reaction (RT-PCR) or other nucleic acid amplification test (NAAT) for test-negative control participants and RT-PCR, NAAT, or antigen tests for enrollment of test-positive case participants. Cases were required to report having at least 1 COVID-19-like symptom in a period of 14 days before or 14 days after the qualifying test. Controls were only required to have a negative diagnostic test. The case-to-control ratio for PREVENT recruitment ranged from 1:1 up to 1:6 based on the number of cases and controls tested each week. In the enrollment survey, participants self-reported demographic information and whether they took time off work.

In this analysis, all HCP who enrolled as COVID-19 cases and worked full time within a health care facility were included. HCP were excluded if they did not report when their symptoms began or whether they took time off of work or not. Some participants enrolled more than once in the PREVENT project after a second or third COVID-19 infection, but we included only the first COVID-19 illness within this analysis. This manuscript follows the Strengthening the Reporting of Observational Studies in Epidemiology (STROBE) reporting guidelines.^10^ This activity was reviewed by the US Centers for Disease Control and Prevention (CDC) and was conducted consistent with applicable federal law and CDC policy.^11^ All participants provided written consent.

### Definitions

HCP who were defined as reporting presenteeism answered no to the survey question, “Did you stop working at any time related to your current/recent illness/exposure (for which you were tested)?” Only full-time HCP were included in the study, and they were defined as those who worked on average more than 36 hours a week within a health care facility (eg, hospital, laboratory, or clinic) and who did not work any of their full-time hours remotely. Of note, the survey did not ask participants who reported presenteeism what the nature of their work became after becoming ill or whether these full-time HCP transitioned to a remote or hybrid role.

HCP were defined as having a recent vaccination if they received an initial 2-dose series of a messenger RNA (mRNA) COVID-19 vaccine, a booster mRNA COVID-19 vaccine, or a seasonal monovalent vaccine dose during the 6 months before their enrollment. The calendar year of the COVID-19 illness was assigned based on the test result date and in the univariate and multivariate analysis was referenced to the first 13 months of the study period (from December 2020 through December 2021). Participants were asked about comorbidities, defined as chronic medical conditions that had been previously diagnosed by a health care practitioner. A list of conditions included as comorbidities is found in eTable 1 in Supplement 1. HCP job roles were categorized a priori based on the degree of patient contact that was anticipated for their job role: substantial patient contact, moderate patient contact, minimal patient contact, or undefined (eTable 2 in Supplement 1).

### Statistical Analysis

To compare characteristics associated with presenteeism, we first summarized characteristics of COVID-19 positive HCP by whether they reported presenteeism or not, including demographic characteristics (eg, age, sex, race [Asian, Black, White, or other, which included American Indian or Alaskan Native and Native Hawaiian or other Pacific Islander], ethnicity [Hispanic or non-Hispanic], educational level, job role, and yearly income) and clinical characteristics (eg, vaccination status, comorbidities, and symptoms). Participants self-reported their race and ethnicity as part of the study survey. Race and ethnicity were assessed to characterize the study population and evaluate for potential differences in outcomes across demographic groups. In our univariate analysis, we compared the frequency of demographic and clinical characteristics in both cohorts, and calculated odds ratios (OR) and 95% CIs for each variable. We then used mixed-effects multivariable logistic regressions with a random effect for month-year to model the odds of presenteeism associated with demographic and clinical characteristics, treated as fixed effects. In this multivariable analysis, we considered 3 different multivariable models. In model A, we evaluated the odds of presenteeism based on demographic variables selected a priori (age, sex, race, ethnicity, education level, and income), patient-contact level, calendar year, and comorbidities, with time period as a random effect. Model B examined the odds of presenteeism based on vaccination status and adjusted for variables in model A. Model C enabled examination of how symptoms were associated with presenteeism and adjusted for variables in model A and B. Variance inflation factor was used to assess collinearity among the covariates.^12^ A Mann-Kendall test was performed without correction to test if there was a monotonic trend in frequency of presenteeism over time. All statistical analysis was completed using R Statistical Software version 4.2.1 (R Project for Statistical Computing).

## Results

### Participant Characteristics

From December 2020 to April 2024, 11 550 HCP at 24 participating academic medical centers were enrolled in the PREVENT project; 7480 were excluded from this analysis due to testing negative for COVID-19, 13 for having an unverified test, and 1265 for not working full time within a health care facility. We excluded 349 for enrolling more than once during the study period. A total of 3721 HCP (2842 [76.4%] aged 18-49 years; 2993 [80.4%] female; 278 [7.5%] Asian, 406 [10.9%] Black, and 2912 [78.3%] White) were evaluated for the analysis (eFigure 1 in Supplement 1). Job roles included nurses (1045 HCP [28.1%]), administrative staff (467 HCP [12.6%]), medical assistants (295 HCP [7.9%]), resident and fellow physicians (245 HCP [6.6%]), and attending physicians (168 HCP [4.5%]), among other roles. The most common comorbidity in participants was body mass index (calculated as weight in kilograms divided by height in meters squared) of 30 or higher (1410 HCP [37.9%]), followed by hypertension (586 HCP [15.8%]) and anxiety (578 HCP [15.5%]). Nine hundred and ninety eight HCP (26.8%) had more than 1 comorbidity. The most commonly reported symptoms among our cohort were fatigue (2567 HCP [69%]), nasal congestion (2449 HCP [65.8%]), and headache (2368 HCP [63.6%]).

### Factors Associated With Presenteeism

Two hundred ninety-three HCP (7.9%) reported presenteeism during their symptomatic COVID-19 illness. When compared with those who stopped working, there was no statistically significant difference in age, sex, race, or ethnicity (Table 1). Presenteeism was less commonly associated with physician residents and fellows (OR, 0.09; 95% CI, 0.03-0.24), nurses (OR, 0.22; 95% CI, 0.15-0.34), medical assistants (OR, 0.26; 95% CI, 0.15-0.48), and advanced practice practitioners (OR, 0.29; 95% CI, 0.13-0.65) compared with those working in administrative or manager roles. When referenced with those who have undergraduate degrees, presenteeism was more likely in those with a graduate or professional degree (OR, 1.98; 95% CI, 1.54-2.53), and compared with those who make less than $50 000 a year, was more likely in those who make more than $100 000 a year (OR, 1.72; 95% CI, 1.16-2.53). When compared with 2020 and 2021, subsequent study period years 2022, 2023, and 2024 were more likely to be associated with presenteeism (OR, 1.34; 95% CI, 0.95-1.89; OR, 1.97; 95% CI, 1.37-2.81; and OR, 2.97; 95% CI, 1.59-5.54, respectively).

**Table 1.** Characteristics of US Health Care Personnel With COVID-19 From December 2020 Through April 2024 Who Stopped Working Compared With Those Who Reported Presenteeism

Characteristic	Participants, No. (%)			OR (95% CI)
	All (N = 3721)	Stopped working (n = 3428)	Reported presenteeism (n = 293)	
Age, y				
18-49	2842 (76.4)	2677 (76.6)	215 (73.4)	1 [Reference]
50-64	809 (21.7)	739 (21.6)	70 (23.9)	1.14 (0.86-1.52)
≥65	70 (1.9)	62 (1.8)	8 (2.7)	1.52 (0.72-3.24)
Sex				
Female	2993 (80.4)	2762 (80.6)	231 (78.8)	1 [Reference]
Male	720 (19.4)	658 (19.2)	62 (21.2)	1.12 (0.84-1.50)
Race				
Asian	278 (7.5)	263 (7.7)	15 (5.1)	0.61 (0.35-1.05)
Black	406 (10.9)	372 (10.9)	34 (11.6)	0.98 (0.67-1.43)
White	2912 (78.3)	2674 (78.0)	238 (81.2)	1 [Reference]
Other^a^	121 (3.3)	115 (3.4)	6 (2.0)	0.55 (0.24-1.26)
Ethnicity				
Non-Hispanic	3319 (89.2)	3061 (89.3)	258 (88.1)	1 [Reference]
Hispanic or Latino	385 (10.4)	352 (10.3)	33 (11.3)	1.10 (0.75-1.61)
Education level				
Undergraduate or technical degree	2264 (60.8)	2127 (62.0)	137 (46.8)	1 [Reference]
Graduate or professional degree	1290 (34.7)	1145 (33.4)	145 (49.5)	1.98 (1.54-2.53)
High school or less	159 (4.3)	148 (4.3)	11 (3.8)	1.14 (0.61-2.16)
Job role				
Administrative or manager	467 (12.6)	394 (11.5)	73 (24.9)	1 [Reference]
Physician (staff or faculty)	168 (4.5)	144 (4.2)	24 (8.2)	0.91 (0.55-1.51)
Physician (resident or fellow)	245 (6.6)	241 (7.0)	4 (1.4)	0.09 (0.03-0.24)
Nurse (RN or LPN)	1045 (28.1)	1003 (29.3)	42 (14.3)	0.22 (0.15-0.34)
Medical assistant, nursing aid, or patient care tech	295 (7.9)	281 (8.2)	14 (4.8)	0.26 (0.15-0.48)
Advanced practice practitioner	135 (3.6)	128 (3.7)	7 (2.4)	0.29 (0.13-0.65)
Research staff	150 (4.0)	125 (3.6)	25 (8.5)	1.04 (0.63-1.72)
Pharmacist or pharmacy personnel	156 (4.2)	134 (3.9)	22 (7.5)	0.86 (0.51-1.45)
Social worker	103 (2.8)	89 (2.6)	14 (4.8)	0.83 (0.45-1.54)
Other staff	957 (25.7)	889 (25.9)	68 (23.2)	0.41 (0.29-0.58)
Yearly income				
<$50 000	567 (15.2)	534 (15.6)	33 (11.3)	1 [Reference]
$50 000-$99 999	1289 (34.6)	1202 (35.1)	87 (29.7)	1.17 (0.78-1.77)
≥ $100 000	1593 (42.8)	1440 (42.0)	153 (52.2)	1.72 (1.16-2.53)
Prefer not to answer	262 (7.0)	242 (7.1)	20 (6.8)	1.37 (0.77-2.43)
Comorbid conditions				
≤1 Condition	2723 (73.2)	2493 (72.7)	230 (78.5)	1 [Reference]
>1 Condition	998 (26.8)	935 (27.3)	63 (21.5)	0.75 (0.55-1.01)
Pulmonary	554 (14.9)	513 (15.0)	41 (14.0)	0.91 (0.65-1.29)
Hypertension	586 (15.8)	531 (15.5)	55 (18.8)	1.23 (0.91-1.68)
Diabetes	190 (5.1)	173 (5.0)	17 (5.8)	1.12 (0.67-1.87)
Immunosuppression	120 (3.2)	115 (3.4)	5 (1.7)	0.52 (0.21-1.28)
Depression/mood disorders	567 (15.2)	537 (15.7)	30 (10.2)	0.60 (0.41-0.89)
Anxiety	578 (15.5)	538 (15.7)	40 (13.7)	0.85 (0.60-1.21)
Body mass index ≥30^b^	1410 (37.9)	1297 (37.8)	113 (38.6)	1.01 (0.79-1.30)
Pregnancy (females)	86 (2.9)	81 (2.9)	5 (2.2)	0.73 (0.29-1.83)
Symptoms				
Fever	1229 (33.0)	1158 (33.8)	71 (24.2)	0.63 (0.48-0.83)
Shortness of breath	611 (16.4)	595 (17.4)	16 (5.5)	0.28 (0.17-0.47)
Cough	2237 (60.1)	2069 (60.4)	168 (57.3)	0.87 (0.68-1.11)
Headache	2368 (63.6)	2211 (64.5)	157 (53.6)	0.64 (0.50-0.81)
Myalgias	1449 (38.9)	1375 (40.1)	74 (25.3)	0.51 (0.39-0.67)
Nausea	627 (16.9)	602 (17.6)	25 (8.5)	0.44 (0.29-0.67)
Nasal congestion	2449 (65.8)	2268 (66.2)	181 (61.8)	0.83 (0.65-1.07)
Fatigue	2567 (69.0)	2402 (70.1)	165 (56.3)	0.55 (0.43-0.71)
Year				
2020 or 2021	1464 (39.3)	1377 (40.2)	87 (29.7)	1 [Reference]
2022	1355 (36.4)	1249 (36.4)	106 (36.2)	1.34 (0.95-1.89)
2023	797 (21.4)	713 (20.8)	84 (28.7)	1.97 (1.37-2.81)
2024	105 (2.8)	89 (2.6)	16 (5.5)	2.97 (1.59-5.54)
Vaccination				
Recently vaccinated	1293 (34.8)	1215 (35.4)	78 (26.6)	1 [Reference]
No recent vaccination	2190 (58.9)	1988 (58.0)	202 (68.9)	1.56 (1.17-2.07)
Partially vaccinated	238 (6.4)	225 (6.6)	13 (4.4)	0.96 (0.51-1.80)
Symptom onset to positive test result, mean (SD), d	2.3 (3.8)	2.3 (3.8)	2.5 (2.7)	1.01 (0.99-1.04)

^a^ Other includes American Indian or Alaskan Native, and Native Hawaiian or other Pacific Islander.

^b^ Body mass index is calculated as weight in kilograms divided by height in meters squared.

While there was no statistically significant difference in the frequency of most individual comorbidities, presenteeism was less likely in HCP with depression or other mood disorders (OR, 0.60; 95% CI, 0.41 to 0.89) compared with those who did not report those mental health comorbidities. Compared with participants who did not report a given symptom, those who reported fever, shortness of breath, headache, myalgias, nausea, or fatigue had significantly lower odds of presenteeism. HCP who were not recently vaccinated were found to have higher odds of presenteeism in the univariate analysis compared with HCP who were (OR, 1.56; 95% CI, 1.17-2.07).

The Figure demonstrates graphically the frequency of presenteeism over time by patient-contact level. The total frequency of presenteeism increased year to year; the frequency was 1 of 27 (1.4%) in 2020, 86 of 1391 (6.2%) in 2021, 106 of 1355 (7.2%) in 2022, 84 of 797 (10.5%) in 2023, and 16 of 105 (15.2%) in 2024.

**Figure.**
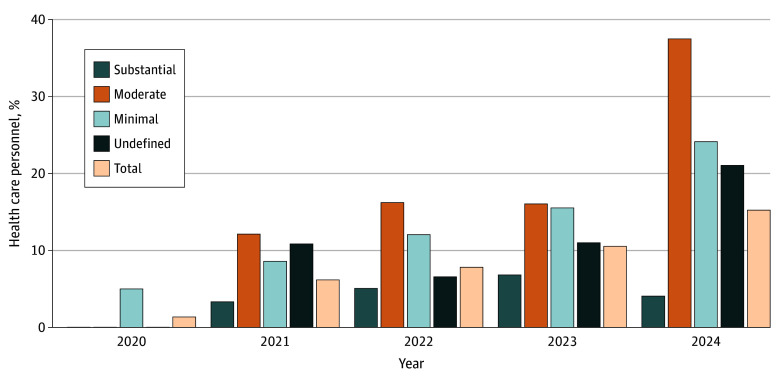
Yearly Percentage of Health Care Personnel With COVID-19 Reporting Presenteeism by Patient Contact Level

### Adjusted Analyses of the Association of Presenteeism With HCP Demographics and Clinical Characteristics

Multivariable logistic regression results for the 3 models are listed in Table 2, with adjusted OR (aOR) values and 95% CIs. In model A, we found no statistical evidence that age and sex were associated with presenteeism. Compared with those with anticipated substantial patient contact, HCP who had minimal, moderate, and undefined patient contact were more likely to report presenteeism (aOR, 3.73; 95% CI, 2.39-4.37; aOR, 3.66; 95% CI, 2.48-5.40; and aOR, 2.67; 95% CI, 1.82-3.93, respectively). HCP with graduate and professional degrees were more likely to report presenteeism compared with those with an undergraduate education level (aOR, 1.90; 95% CI, 1.45-2.50), and HCP whose yearly income was more than $100 000 were more likely to report presenteeism than those who made less than $50 000 a year (aOR, 1.74; 95% CI, 1.12-2.69). Calendar year was also associated with presenteeism in our adjusted analysis, with years 2023 and 2024 associated with presenteeism compared with the first 13 months of the study, 2021 and 2022 (aOR, 1.69; 95% CI, 1.16-2.48 and aOR, 2.72; 95% CI, 1.41-5.23, respectively).

**Table 2.** Association of Participant Characteristics With Presenteeism Among US Health Care Personnel With COVID-19 From December 2020 Through April 2024^a^

Characteristic	aOR (95% CI)		
**Model A** ^a^	**Model B** ^b^	**Model C** ^c^
Calendar period			
2020 or 2021	1 [Reference]	1 [Reference]	1 [Reference]
2022	1.25 (0.87-1.80)	1.16 (0.80-1.69)	1.15 (0.81-1.62)
2023	1.69 (1.16-2.48)	1.52 (1.02-2.27)	1.54 (1.05-2.26)
2024	2.72 (1.41-5.23)	2.47 (1.27-4.80)	2.28 (1.20-4.35)
Age			
18-49 y	1 [Reference]	1 [Reference]	1 [Reference]
50-64 y	1.06 (0.79-1.43)	1.05 (0.78-1.41)	0.99 (0.73-1.34)
≥65 y	1.42 (0.65-3.07)	1.39 (0.64-3.01)	1.26 (0.57-2.77)
Sex			
Female	1 [Reference]	1 [Reference]	1 [Reference]
Male	0.97 (0.72-1.32)	0.97 (0.71-1.31)	0.92 (0.68-1.26)
Ethnicity			
Hispanic	1.17 (0.78-1.74)	1.17 (0.79-1.75)	1.24 (0.83-1.86)
Non-Hispanic			
Job role			
Minimal contact	3.23 (2.39-4.37)	3.23 (2.39-4.37)	3.01 (2.22-4.09)
Moderate contact	3.66 (2.48-5.40)	3.65 (2.47-5.39)	3.37 (2.27-5.01)
Substantial contact	1 [Reference]	1 [Reference]	1 [Reference]
Undefined contact	2.67 (1.82-3.93)	2.67 (1.81-3.93)	2.59 (1.75-3.83)
>1 Comorbidity	0.89 (0.64-1.24)	0.90 (0.64-1.25)	0.98 (0.70-1.36)
Education level			
High school or less	0.93 (0.48-1.80)	0.95 (0.49-1.84)	0.94 (0.49-1.83)
Undergraduate degree	1 [Reference]	1 [Reference]	1 [Reference]
Graduate or professional degree	1.90 (1.45-2.50)	1.88 (1.43-2.48)	1.78 (1.35-2.35)
Income			
<$50 000	1 [Reference]	1 [Reference]	1 [Reference]
$50 000-$99 999	1.20 (0.78-1.86)	1.21 (0.78-1.86)	1.19 (0.77-1.85)
≥$100 000	1.74 (1.12-2.69)	1.73 (1.12-2.68)	1.68 (1.08-2.62)
Prefer not to answer	1.31 (0.72-2.40)	1.31 (0.72-2.39)	1.29 (0.70-2.36)
Vaccination status			
No recent vaccination	NA	1.29 (0.96-1.74)	1.23 (0.91-1.66)
Recent vaccination	1 [Reference]	1 [Reference]	1 [Reference]
Partially vaccinated	NA	1.08 (0.57-2.04)	1.05 (0.56-1.97)
Symptoms			
Fever	NA	NA	0.83 (0.61-1.12)
Shortness of breath	NA	NA	0.37 (0.21-0.63)
Cough	NA	NA	1.25 (0.96-1.63)
Headache	NA	NA	0.90 (0.69-1.18)
Myalgias	NA	NA	0.74 (0.54-1.00)
Nausea	NA	NA	0.68 (0.44-1.06)
Nasal congestion	NA	NA	1.03 (0.79-1.34)
Fatigue	NA	NA	0.77 (0.59-1.02)

Abbreviation: NA, not applicable.

^a^ Model A adjusted for age, sex, ethnicity, education level, income, patient contact level, calendar year, and comorbidities, with time period as random effect.

^b^ Model B adjusted for age, sex, ethnicity, education level, income, patient contact level, calendar year, comorbidities, and vaccination status, with time period as random effect.

^c^ Model C adjusted for age, sex, ethnicity, education level, income, patient contact level, calendar year, comorbidities, vaccination status, and each variable of interest, with time period as random effect.

Model B evaluated the association of vaccination status and presenteeism and did not find evidence for an association in vaccination status and HCP who reported presenteeism. Model C examined the association of HCP reporting specific symptoms during their COVID-19 illness and presenteeism and found that those who reported shortness of breath were less likely to report presenteeism (aOR, 0.37; 95% CI, 0.21-0.63).

## Discussion

This study identifies the frequency and factors associated with presenteeism in HCP with symptomatic COVID-19. Nearly 8% of HCP in the cohort reported that they did not stop working during their COVID-19 illness. We found that later calendar years of the study period (2023-2024) were more greatly associated with presenteeism, with the frequency of presenteeism increasing to 15.2% in 2024. HCP who were anticipated to work less directly with patients were associated with presenteeism, as were HCP with a higher income level and higher education level. Conversely, HCP that reported shortness of breath during their illness were associated with lower odds of presenteeism.

Evidence suggests that presenteeism in HCP has been a common practice for other respiratory illnesses. LeVela et al^13^ surveyed HCP in 2007 and 86% reported that they attended work while symptomatic with influenza-like illness (ILI) symptoms. Another cross-sectional survey of HCP in 2020 found similar rates, with 89% of HCP reporting that they typically worked with nonfebrile “minor” ILI symptoms (eg, sore throat, congestion, cough, and fatigue).^14^ Previous reports on COVID-19 presenteeism offer insight into its prevalence. In a review of HCP COVID-19 presenteeism in 327 workers at a Veteran Affairs (VA) health care system from December 2020 to September 2021, the overall frequency was 49.8%.^15^ In that study, presenteeism included HCP who reported working any number of days while symptomatic, but may have stopped for part of their illness. In our study, the frequency of presenteeism was comparatively lower (7.9%), but we included only those who reported that they did not stop working at all during their COVID-19 illness. It is likely that our overall frequency of presenteeism would increase if we also captured those who did continue working for only a portion of their symptomatic illness before ultimately leaving work after testing positive. For this study, we chose to examine those individuals who had confirmed symptomatic COVID-19 and reported that they never stopped working.

The variable we examined that had the greatest association with presenteeism was the calendar year that the HCP became ill. In later years—2023 through 2024—HCP were more likely to report presenteeism. The Figure demonstrates this as well, showing how presenteeism increases each year over the course of the pandemic. What started out as relatively low frequency in 2020 (1.4%) steadily increased in nearly all patient contact levels. By 2024 the overall frequency was 15.2%.

A combination of factors may have contributed to the rising frequency of presenteeism. Increasing population immunity and decreasing virulence of later COVID-19 variants reduced the severity of the illness, and HCP may have perceived less danger to others while working with COVID-19.^16^ Later federal guidelines reduced the total time required for isolation when ill, and participants who tested positive may have already completed their recommended isolation period and had no need to take time off work.^17^ The rising prevalence of remote work may have also contributed.^18^ While all of the HCP who enrolled in the study were working on-site in a health care facility at the time of testing positive, the proportion who transitioned to remote work may have increased over time as this became a more acceptable option for some HCP.

Presenteeism was overall more likely in those who worked in fields with minimal and moderate patient contact, which could indicate that there was less concern of HCP-to-patient transmission in these roles. Those who work less closely with patients may have had more opportunities to isolate at work, less fear that their illness would result in an iatrogenic patient infection, or had an easier transition to remote work. There may also have been a disparity in benefits between those who had substantial patient contact—primarily physicians and nurses in our study—and those that did not. The surveys used did not specifically ask about sick days, so it is unclear who had access to those benefits and how this factored into the decision to continue working. It’s important to consider whether hospital support staff that did not participate in patient care had equal access to sick days, which could have impacted the decision to continue working.

### Limitations

Our study was limited to HCP in academic medical centers who were predominantly White and female and may not reflect presenteeism in other populations. The internal validity was also dependent on reliable self-reporting from HCP who completed the survey after enrollment. Results may be subject to recall bias or social desirability bias (the general workplace acceptance of presenteeism may have changed over time). Additionally, the main question that we used to designate HCP presenteeism was “Did you stop working at any time related to your current/recent illness/exposure (for which you were tested)?” This indirect line of questioning was not originally intended in the PREVENT study to identify presenteeism specifically, and a more direct line of questioning may have revealed a higher rate among HCP.

The PREVENT project data included demographics, clinical characteristics, and COVID-19 test timing, but did not survey participants on their rationale for presenteeism and what drove their behavior, which would be helpful in understanding ways to mitigate the problem going forward. In the study by Linsenmeyer et al^16^ of the VA health care system, common reasons for presenteeism were “knowing how to take precautions at work to avoid getting others sick” (67%), “concerns over workload burden for coworkers” (66%), and “personal responsibility” (45%). We also did not specifically assess access to paid sick leave and the nature of their work while ill. Some may have continued working remotely, making the risk of workplace transmission difficult to estimate; but many job roles required either direct patient care (eg, nurses and medical assistants) or physical presence (eg, maintenance, pharmacy, custodial, and desk clerks) and were not always immediately adaptable to telework (see eTable 2 in Supplement 1). We considered that the overall increase in telework within health care in the last 4 years may have contributed to an overall increase of reported presenteeism over time in our study.^19^

## Conclusions

In conclusion, in a cohort of HCP with symptomatic COVD-19, we found that a substantial proportion reported presenteeism, with an increasing frequency over time. Calendar year of testing, patient contact level, education, and income were associated with presenteeism. HCP who reported the symptom of shortness of breath had a negative association with presenteeism. These variables help us understand the degree of presenteeism among HCP and the characteristics of those who report it. Further studies are needed to understand the rationale behind presenteeism in HCP and clarify why the frequency increased over time.

## Notes

Supplement 1. eFigure. Flow Chart of Participants in Study of Presenteeism Among US Health Care Personnel (HCP) With COVID-19 From December 2020 Through April 2024 eTable 1. All Comorbid Conditions Included in the Study eTable 2. Health Care Personnel Job Roles With Presenteeism by Patient Contact Level eAppendix. Additional Contributions

Supplement 2. Data Sharing Supplement
