# Supplementary material for: Presenteeism Among Health Care Personnel With COVID-19
Source: JAMA Netw Open. 2025 Dec 3;8(12):e2546405. doi: 10.1001/jamanetworkopen.2025.46405 (PMC12676355; doi:10.1001/jamanetworkopen.2025.46405)
Supplement: Supplement 1. — eFigure. Flow Chart of Participants in Study of Presenteeism Among US Health Care Personnel (HCP) With COVID-19 From December 2020 Through April 2024 eTable 1. All Comorbid Conditions Included in the Study eTable 2. Health Care Personnel Job Roles With Presenteeism by Patient Contact Level eAppendix. Additional Contributions [file jamanetwopen-e2546405-s001.pdf]

Supplemental Online Content

Crosby JC, Leon ES, Chinnock B, et al. Presenteeism among health care personnel with COVID-19. *JAMA Netw Open*. 2025;8(12):e2546405.
doi:10.1001/jamanetworkopen.2025.46405

eFigure. Flow Chart of Participants in Study of Presenteeism Among US Health Care Personnel (HCP) With COVID-19 From December 2020 Through April 2024
eTable 1. All Comorbid Conditions Included in the Study
eTable 2. Health Care Personnel Job Roles With Presenteeism by Patient Contact Level

This supplemental material has been provided by the authors to give readers additional information about their work.

© 2025 Crosby JC et al. *JAMA Network Open*.

eFigure. Flow Chart of Participants in Study of Presenteeism Among US Health Care Personnel (HCP) With COVID-19 From December 2020 Through April 2024

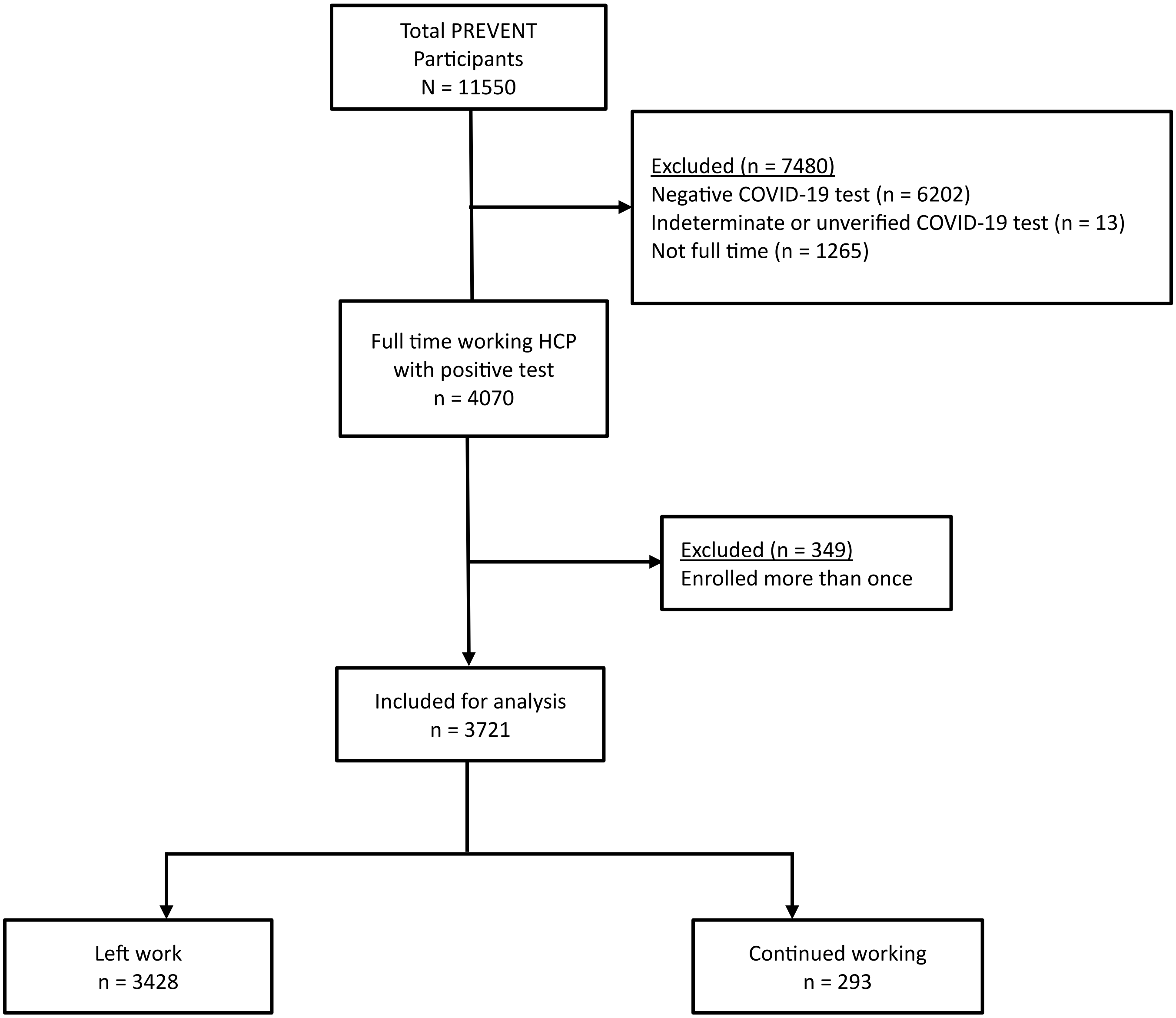

© 2025 Crosby JC et al. *JAMA Network Open*.

eTable 1. All Comorbid Conditions Included in the Study

Stroke
Diabetes mellitus, type I
Diabetes mellitus, type II
Diabetes mellitus, unspecified type
Chronic kidney disease
Dialysis
Solid organ transplant (kidney, liver, lungs, heart)
Hematopoietic stem cell transplant
Autoimmune or rheumatologic disease
Other immunosuppressing condition
Active cancer
Deep vein thrombosis or pulmonary embolism
Chronic liver disease
Depression or other mood disorder
Anxiety/obsessive- compulsive/trauma or stressor related disorder
Other mental health condition
Movement or motor disorders
Alcohol use disorder
Sleep disorder
Cognitive/neurodevelopmental disorder

© 2025 Crosby JC et al. *JAMA Network Open*.

eTable 2. Health Care Personnel Job Roles With Presenteeism by Patient Contact Level [a]

Job Role	N=[a] (%)
**Substantial Patient Contact**	**N= 95 (32.4%)**
Nurse - Registered nurse	41 (43.2)
Physician - Staff/Faculty	24 (25.3)
Medical Assistant	10 (10.5)
Advanced Practice Provider - Nurse Practitioner	6 (6.3)
Nursing Aide/Nursing Assistant/Patient Care Technician	4 (4.2)
Physician - Intern/Resident	3 (3.2)
Advanced Practice Provide Physician Assistant	2 (2.1)
Paramedic/Emergency Medical Technician	2 (2.1)
Nurse - Licensed Practical Nurse	1 (1.1)
Physical Therapist or Assistant	1 (1.1)
Physician - Fellow	1 (1.1)
Respiratory Therapist or Assistant	1 (1.1)
Home Health Aide/In-home Caregiver	0 (0)
Patient Care Technician/Nursing Aide/Nursing Assistant	0 (0)
Occupational Therapist or Assistant	0 (0)
Phlebotomist	0 (0)
Speech Therapist or Assistant	0 (0)
**Moderate Patient Contact**	**N=46 (15.7%)**
Research Staff (clinical, translational, or basic science)	25 (54.3)
Social Worker	14 (30.4)
Environmental Services/Custodial/Housekeeping Staff	3 (6.5)
Dietician/Nutritionist	3 (6.5)
Food Service/Cafeteria Staff	1 (2.2)
Chaplain	0 (0)
**Minimal Patient Contact**	**N=108 (36.9%)**
Administrative Staff/Managers	71 (65.7)
Pharmacist/Pharmacy Personnel	22 (20.4)
Clerk/Registration staff	12 (11.1)
Laboratory Personnel	5 (4.6)
Facilities/Maintenance	1 (0.9)
**Undefined Patient Contact**	**N=44 (15.0%)**
Other	28 (63.6)
Missing	9 (20.5)
Information Technology/Computer Support	5 (11.4)
Health Sciences Students (medical, nursing, pharmacy, dentistry, advanced practice provider, or others)	2 (4.5)
Security Personnel	0 (0)
Volunteer	0 (0)

a. Some participants indicated more than one job role

© 2025 Crosby JC et al. *JAMA Network Open*.
