# Supplementary material for: Presenteeism Among Health Care Personnel With COVID-19
Source: JAMA Netw Open. 2025 Dec 3;8(12):e2546405. doi: 10.1001/jamanetworkopen.2025.46405 (PMC12676355; doi:10.1001/jamanetworkopen.2025.46405)
Supplement: Supplement 2. — Data Sharing Supplement [file jamanetwopen-e2546405-s002.pdf]

Data Sharing Statement

Crosby. Presenteeism Among Health Care Personnel With COVID-19. *JAMA Netw Open*. Published December 02, 2025. doi:10.1001/jamanetworkopen.2025.46405

Data
Data available: Yes
Data types: Deidentified participant data
How to access data: https://www.emergencyidnet.org/current-research/prevent-project-20
When available: With publication

Supporting Documents
Document types: None

Additional Information
Who can access the data: Data is publicly available to anyone requesting it
Types of analyses: For any purpose, excluding the following: no findings related to the primary vaccine effectiveness objective may be published, presented, or released by the PREVENT investigators without approval from the CDC COVID-19 Vaccine Task Force investigators. The primary objective of the PREVENT project is to evaluate SARS-CoV-2 vaccine effectiveness in preventing laboratory-confirmed symptomatic COVID-19 among HCP, including the effect of vaccine boosters and temporal changes in vaccine effectiveness.
Mechanisms of data availability: Without investigator support
